# Voyages in Vacation.—IX

**Published:** 1888-11-24

**Authors:** 


### VOYAGES IN VACATION.—IX.

BY ONE IN SEARCH OF RESTFUL RESTORATIVES.

*(Continued from page 103.)*

### IMPRESSIONS OF ST. PETERSBURG.

We omitted to mention an incident which occurred at Cronstadt which may convey a useful warning to others. As the *Victoria* cast anchor, we found ourselves within a short distance of a Hull steamer—one of the Wilson line. The Custom House officers were then inspecting her, and we observed some commotion on board, followed by continuous cheering. It turned out that one of the passengers had been foolish enough to try and enter Russia without a passport. He was accordingly taken in charge by the police, put into a boat, and carried off to the shore. This incident accounted for the cheers we had heard, which were given by the passengers as he left the steamer. Luckily for him he had a brother at St. Petersburg, who was able to secure his release after a few hours' detention; otherwise the adventure might have proved more exciting than agreeable.

So soon as dinner was over we debated whether we would go on shore that evening or not. Three only of the passengers ultimately went, one to stay at an hotel and the other two to procure a guide. To those who have travelled much it sounds ridiculous to talk of a guide to any place. We found, however, as a matter of experience, that a courier who knows the language is as essential to a successful visit and journey through Russia as steam is to the steam-engine. There can be no doubt that the Russians have no great liking for the English. On the contrary, they regard them with suspicion, which is often quickened into dislike on the slightest provocation, to be followed by a rough rudeness which a stranger cannot easily overlook. A knowledge of Russian is therefore essential, as it often happens that a Russian who knows English and can understand all that you are saying, will deny all knowledge of the language, and declare he only understands his own tongue and a little German. Then, again, few visitors will find the climate of St. Petersburg conducive to their comfort, except, perhaps, for a short period in the winter, as the damp and swampy character of the place not infrequently sets up internal inflammation after a brief residence, and speedily frightens most strangers away. The atmosphere is no doubt beautifully clear, and the whole aspect of the city is bright and enticing, but somehow the climate proves depressing to strangers, who usually hurry away after a fortnight's stay. For these and other reasons those who are wise will engage a courier at once, and then proceed, with his assistance, to map out their days, so as to embrace all the chief places and points of interest. Unfortunately, good couriers are very scarce, and, so far as we could learn, there is only one who really speaks English, and can be thoroughly trusted to give you good value for your money. His name is Mr. Charles Kuntzé, 66, Perspective de Nevsky, and anyone who is fortunate enough to secure his services will find Russia full of interest in many ways. Mr. Kuntzé will take entire charge of a party, bag and baggage, and as he not only knows every nook and corner of the towns he undertakes to show, but is everywhere known to the officials, there will be no difficulty in getting to see everything of interest with the greatest expedition under his guidance. From our experience of Russia we have no hesitation in declaring that, apart from the impossibility of seeing a place thoroughly, the saving in expense and time which the services of a good courier secure, is so material as to render it impossible for any reasonably prudent person to attempt to see Russia without engaging Mr. Charles Kuntzé or one of his class.

Before leaving home a friend had declared emphatically that St. Petersburg and Moscow were the two cities of Europe to be seen after all others had been visited, because after them there was very little left to be desired in the way of populous places. We were curious, therefore, to see whether the favourable impressions we had gained by approaching St. Petersburg from the sea would be maintained on a closer inspection or not. It was a beautiful, warm, sunny morning when we left the *Victoria* and crossing over St. Nicholas Bridge, entered the more fashionable and central portion of the city. Everywhere fine buildings, wide streets, and squares were encountered, which could not fail to impress the stranger favourably. One of the first things which struck us was the fact that everybody wore a long top-coat, which was kept fastened despite the briliant sunshine. This caused some surprise until we found ourselves on the shaded sides of the streets, where the cold was keen and piercing. We were thus taught to follow the native custom, and to wear a great-coat on all occasions and throughout the day. Some who failed to take these precautions had reason to repent their temerity, and suffered no little pain and discomfort in consequence. A stranger is able to stand the climate fairly well for the first week, after which time he is not infrequently laid up with an attack of what the residents call "typhus," *i.e.*, great pain, often accompanied by inflammation of the bowels, with a high temperature and the usual attendant discomforts. The contrast between the climate of St. Petersburg and Moscow is most marked. The former city, built upon a marsh, is swampy, damp, and chilling to an extent we have never before experienced; whilst Moscow is warm, dry, invigorating, and health-restoring to a remarkable degree. The effects of the two climates upon one of our party were astonishing. At St. Petersburg he was more or less worried by an asthmatical and troublesome cough, with much throat trouble, but at Moscow he was free from both affections, and quite a different man. In the same way all intestinal inflammation disappeared during our visit to Moscow. Of course very many of the better-class residents leave St. Petersburg for the country during several months in each year, the winter being the favourite and most healthy period for all classes.

The streets, which, as we have said, are well-arranged and imposing from their great width, have one serious drawback—petrified kidneys, which constitute the usual form of pavement for roadways and side-walks, so that the discomforts attending locomotion may be better imagined than described. Here and there short lengths of wood pavement have been put down, but speaking generally, it may be stated that all Petersburg is paved with stones, and this fact is jolted, shaken, and driven into the mind of the visitor at the very outset, to his no small discomfort and disgust. Hence, a word may profitably be said on the subject of carriages. There are droschkies in as great quantities as there are hansom cabs in London, and there, as here, the popular vehicles differ in quality to an equal extent. In the commonest droschkies one can drive for a mile for about fourpence, but double that sum will secure a superior vehicle with indiarubber tyres, a splendid horse, and a smart appearance. The increased cleanliness of the latter also is worth more than half the money. The droschky is very low, with four wheels, those in front being very small, and the traces are fixed to their axles. The driver, or isvostchik, sits in front on a raised seat, he is dressed in a long robe, usually of blue cloth, with an embroidered sash or belt of many colours round his waist. He holds the reins in both hands, has no whip, but has a small lash or piece of whipcord attached to the ends of the reins, which he uses freely as occasion may require. There is no back to the droschky, and it is usual to see the lady supported by the arm of the gentleman who accompanies her, a practice which has a novel effect, but appears to be highly popular. There are leather aprons for wet weather, and a few droschkies have hoods, but these are only used in very bad weather, and are not usually met with. In some respects the droschky resembles a Victoria, though it is much smaller, lighter, and far less comfortable in every way. There are, of course an abundance of carriages obtainable, which are not only very comfortable but most moderate in cost. A pair-horse carriage, excellently found in every way, can be hired for the whole day for from one pound to twenty-five shillings. A close carriage without indiarubber tyres is quite maddening, owing to the stones, and no one who has had experience of such a drive would repeat it for any consideration whatever.

*(To be continued.)*

